# Conservative management of endometrial stromal sarcoma at stage III: A case report

**DOI:** 10.3892/ol.2014.2288

**Published:** 2014-06-26

**Authors:** RUIYING DONG, HONGLUAN MAO, PEIHAI ZHANG

**Affiliations:** Department of Obstetrics and Gynecology, Qilu Hospital of Shandong University, Jinan, Shandong 250012, P.R. China

**Keywords:** endometrial stromal sarcoma, conservative management, progestin therapy

## Abstract

Endometrial stromal sarcoma (ESS) is a rare malignant tumor of the uterus. The standard treatment is surgery, such as total hysterectomy with bilateral salpingo-oophorectomy. The use of adjuvant treatment, including chemotherapy, radiation therapy and endocrine therapy, remains controversial, so it is uncommon for conservative management to be performed in patients with low-grade ESS. The present study reports the case of a 19-year-old female with ESS at stage III who underwent a local mass resection by laparoscopic surgery. A high dose of progestin (medroxyprogesterone acetate) therapy was then administered. Conservative management resulted in complete remission of the low-grade ESS, with no sign of recurrence at the 33-month follow-up.

## Introduction

Endometrial stromal sarcoma (ESS) is a rare malignant tumor of the uterus, which accounts for <2% of all uterine malignancies and approximately one-fifth of all uterine sarcomas (1,2). ESS can be classified into low and high grade according to the tumor characteristics. However, as high-grade ESS neither shows endometrial stromal differentiation nor expresses estrogen receptor (ER) or progesterone receptor (PR), only low-grade ESS is currently considered as ESS, whereas high-grade ESS is known as undifferentiated endometrial sarcoma (3). In general, ESS occurs in perimenopausal females (4). The standard treatment is surgery, such as total hysterectomy with bilateral salpingo-oophorectomy. The role of adjuvant treatment, which includes chemotherapy, radiation therapy and endocrine therapy, remains controversial. Additionally, the probability of ESS relapse remains at ~50%.

Due to a significantly improved prognosis compared to other uterus sarcomas, several cases of fertility-preserving surgery have been reported for patients with ESS (4–7). However, the majority were patients at stage I. The current study presents a case of ESS at stage III that was treated by local mass resection and uterine reconstruction, and subsequent adjuvant treatment. To date, the patient has been followed up for 33 months and there is no sign of recurrence. Patient provided written informed consent.

## Case report

A 19-year-old female (gravida 0, para 0) was admitted to the Qilu Hospital of Shandong University (Jinan, Shandong, China) following one day of acute hypogastralgia. A B ultrasound examination demonstrated a heterogenous echo (9.1×7.5 cm) in the anterior wall of the uterus. The bilateral annex area was normal. Upon physical examination, tenderness of the lower abdomin was apparent, but with no rebound tenderness. A rectal examination indicated an enlarged uterus (pregnant uterus of ~3 months gestation in size) with significant pain in the anterior wall. No adnexal mass was palpatable. The patient was not sexually active and presented with a normal menstrual history. The primary diagnosis was of degeneration of a myoma of the uterus.

**Figure 1:**
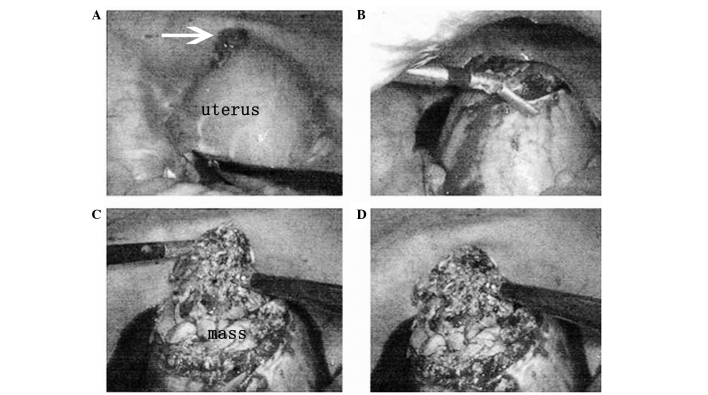
Pre-operative and intraoperative findings indicating an intramural mass on the anterior wall of the uterus. (A and B) Pre-operative image showing the tumor breaking with necrosis inside (white arrow). (C and D) Intraoperative image revealing fragile yellow and ropy tumor tissues without a clear margin.

Subsequent to two days of adjuvant examinations, a laparoscopy was performed. Upon surgical exploration, 200 ml of a faint yellow effusion was found in the pelvic cavity, and an enlarged uterus (pregnant uterus of ~3 months gestation in size) and severe adhesion between the anterior wall of the uterus, omentum majus and intestinal canal was observed. As shown in Fig. 1A and B, following the separation of the adhesion, the anterior wall of the uterus looked full and convex, and a 2-cm broken sore with endometrioid necrosis inside existed in the anterior serosa. The ovaries were normal. Subsequent to resecting the serosa on the anterior wall of the uterus, an intramural mass was found with unclear margins. The tumor was fragile, with a yellow and ropy appearance, with hemorrhage and necrosis inside (Fig. 1C and D). The mass (10×9 cm) was resected from the anterior wall of the uterus body, and histological examination of a frozen section of the resected mass obtained during the surgery indicated a low-grade ESS. As the patient’s family was adamant with regard to preserving the fertility of the patient, a decision was made to preserve the uterus, and a subsequent laparotomy was performed. A fusiform incision of the myometrium was created ~1 cm lateral to the former uterus incision. Total resection of the mass was achieved and the uterus was reconstructed. A 5×4-cm sheet and thickened region of the intestinal canal was adherent to the anterior wall of the uterus, with a 2×1-cm hemorrhagic and necrotic nodule on the surface. Therefore, the surface nodule and partial adhesive omentum majus were removed. No other abnormalities were found during the following abdominal and pelvic exploration.

**Figure 2:**
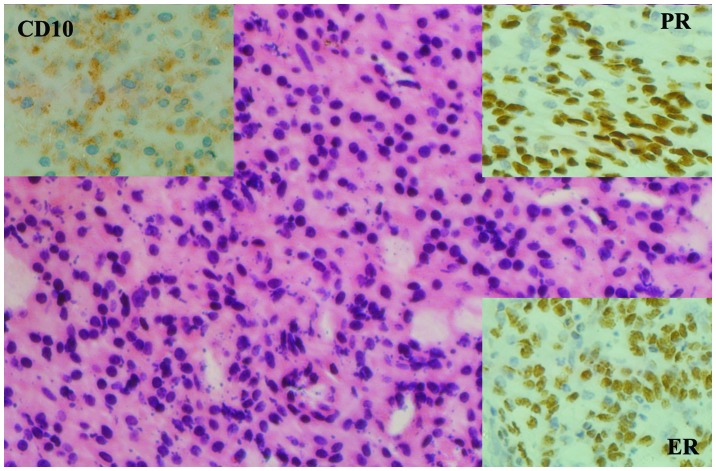
Immunohistochemical analysis of cluster of differentiation (CD)10, estrogen receptor (ER) and prgesterone receptor (PR) expression in endometrial stromal sarcoma (ESS). CD10 and ER expression was low or moderate in ESS, whereas PR expression was moderate or high.

The post-operative pathohistological analysis of a paraffin-embedded section showed a low-grade ESS in accordance with the previous pathological diagnosis of the frozen section. Nodules from the surface of the intestine had a small quantity of endometrial stromal sarcoma cells. No sarcoma cells were found in the omentum majus. The staining intensity was defined as follows: −, negative; +, weak; ++, moderate; and +++, strong. As shown in Fig. 2, the immunohistochemistry assay showed the following results: Cluster of differentiation (CD)10(+), α-inhibin(−), CD34(−), smooth muscle actin(+/−), ER(++) and PR(+++).

Post-operatively, the patient was administered 250 mg medroxyprogesterone acetate daily for 1 year to inhibit tumor recurrence. Sequential clinical examinations and radiographical studies have been used in post-operative surveillance, and a 33-month follow-up examination showed no signs of recurrent disease.

## Discussion

ESS is divided into two subtypes, high-grade ESS and low-grade ESS, with totally different prognoses. High-grade ESS has a relatively poor prognosis, whereas the prognosis of low-grade ESS is relatively favorable. The significant factors affecting the treatment outcome include clinical stage, histological subtype, cell differential degree, tumor size and expression of sexual hormone receptors (8). The common clinical manifestation is abnormal vaginal bleeding. In the current case, the patient presented with acute hypogastralgia as the initial symptom, which was believed to be caused by the large tumor size and uterus perforation on the basis of previous surgical situations.

The main surgery for ESS is total abdominal hysterectomy with adnexectomy. However, with regard to the surgical options for young patients, further studies are required to analyze the feasibility of fertility preservation. Several cases have previously reported that fertility-preserving treatment for ESS is feasible (4–7). However, the majority of these patients were at stage I. In the present case, the local mass was as large as 10 cm with a broken sore on the surface, and the adhesion with the omentum majus and intestine was confirmed to exhibit metastasis. Taking all these facts into account, the tumor was classified as clinical stage III. As the patient was only 19-years-old, a local mass resection and uterine reconstruction were performed to preserve fertility. Post-operatively, endocrine treatment was commenced. To date, the patient is well without any evidence of recurrence following a 33-month follow-up period. It has been reported that the median time to recurrence is 65 months for stage I ESS and 9 months for stages III–IV (9). Therefore, follow-up is necessary for those patients with ESS who have undergone fertility-preserving treatment in order to identify and treat recurrence at an early stage. To date, there have been three reported cases of patients with ESS who experienced a successful pregnancy following fertility-preserving treatment (4–6). Koskas *et al* reported the case of a 34-year-old female treated conservatively for low-grade ESS (LGESS) who conceived rapidly following hysteroscopic resection of the tumor. However, in the postpartum period, pelvic pain motivated a laparoscopic exploration, which revealed severe peritoneal recurrence (6). It is postulated that changes in hormone levels during pregnancy enhanced the process of ESS. This case indicated that pregnancy may contribute to the development of LGESS. Therefore, the decision to preserve fertility and undergo pregnancy should be taken into consideration.

Adjuvant treatments for LGESS consist of chemotherapy, radiotherapy and endocrine therapy. Based on experience from clinical practice that has shown that LGESS is generally a hormonally-sensitive tumor with indolent growth, adjuvant progestin treatment is currently the most effective treatment for treatment of LGESS and should be considered as a routine adjuvant therapy for the treatment of ESS or recurrent ESS, particularly for those with strong positivity for PR staining (10,11). The present study patient with stage III ESS showed no signs of recurrence following conservative treatment, including fertility-preserving surgery and high-dose progestin treatment. Additionally, it is known that adjuvant aromatase inhibitors may also aid in the treatment of ESS, and that the combined application of progestin and aromatase inhibitors may have future development potential for ESS treatment; these topics have already been the subject of studies and reviews (12–14).

As the prognosis of LGESS is usually favorable, conservative surgery is a logical intervention for young nulliparous females. However, the significant prognostic factors affecting treatment must be taken into consideration. Conservative treatment for LGESS has not been experienced a great deal clinically and is problematic, as no randomized trials are available to offer a reliable theoretical basis. Overall, the decision of whether to administer conservative management for young females with LGESS should be taken according to the individual clinical condition, and further studies are required.
